# Formononetin promotes angiogenesis through the estrogen receptor alpha-enhanced ROCK pathway

**DOI:** 10.1038/srep16815

**Published:** 2015-11-16

**Authors:** Shang Li, Yuanye Dang, Xuelin Zhou, Bin Huang, Xiaohui Huang, Zherui Zhang, Yiu Wa Kwan, Shun Wan Chan, George Pak Heng Leung, Simon Ming Yuen Lee, Maggie Pui Man Hoi

**Affiliations:** 1 State Key Laboratory of Quality Research in Chinese Medicine and Institute of Chinese Medical Sciences, University of Macau, Macao, China; 2 School of Biomedical Sciences, Faculty of Medicine, The Chinese University of Hong Kong, Shatin, N.T., Hong Kong, China; 3 State Key Laboratory of Chinese Medicine and Molecular Pharmacology, Department of Applied Biology and Chemical Technology, The Hong Kong Polytechnic University, Hong Kong, China; 4 Pharmacology and Pharmacy, Faculty of Medicine, The University of Hong Kong, Hong Kong, China

## Abstract

Formononetin is an isoflavone that has been shown to display estrogenic properties and induce angiogenesis activities. However, the interrelationship between the estrogenic properties and angiogenesis activities of formononetin are not well defined. In the present study, docking and enzymatic assay demonstrated that formononetin displayed direct binding to the ligand-binding domain (LBD) of estrogen receptor alpha (ERα) with an agonistic property. Results from Human Umbilical Vein Endothelial Cells (HUVEC) by using real-time migration xCELLigence system, immunofluorescence and western blotting provided strong evidences of formononetin induced endothelial cell migration and dramatic actin cytoskeleton spatial modification through ERα-enhanced-ROCK-II/MMP2/9 signaling pathways. In addition, results from co-immunoprecipitation suggested formononetin induced cell migration via recruiting of ERα/ROCK-II activated complex formation. More interestingly, in zebrafish embryo we observed that formononetin significantly promoted angiogenic sproutings in the subintestinal vessels (SIVs) that could be completely abolished by ROCK inhibitor. In this study, we elucidated the underlying mechanisms that formononetin produced proangiogenesis effects through an ERα-enhanced ROCK-II signaling pathways. Results from the present study also expand our knowledge about the enigmatic underlying mechanisms of phytoestrogenic compounds in the promotion of angiogenesis in relation to ERα and ROCK interaction in endothelial cells and their relationship with actin assembly and cell migration.

Many flavonoids have been shown to possess pro-angiogenic properties^1^,^2^,^3^,^4^, but the underlying mechanisms are incompletely understood. A number of studies have shown that formononetin, an isoflavone and phytoestrogen that displays pro-angiogenic effects, could promote vascular recovery by regulating endothelial cell proliferation and migration at sites of vascular injury^5^,^6^. It has been reported that, formononetin in a rat fracture model promoted early fracture healing through stimulating angiogenesis by up-regulating VEGF and Flk-1^5^ and formononetin promoted the recovery of the migration and proliferation of wounded (human umbilical vein endothelial cells) HUVEC via increased levels of growth factors^6^. Estrogen receptors (ERs) are present in the vascular endothelium and it is observed that estradiol could induce angiogenesis through the activation of both long-term (genomic) and rapid (non-genomic) ER signaling^7^,^8^,^9^. While both genomic and non-genomic ER signaling are involved in angiogenesis and re-vascularization, it has been observed that exposure to the ER agonist 17β-estradiol (E2) leads to ERα membrane translocation and rapidly affect membrane modifications^8^,^9^, suggesting fast modifications of actin cytoskeleton closely related to the non-genomic actions of ERα^10^. The dynamics of actin cytoskeleton and stress fiber formation are known to be regulated by the small GTPase protein RhoA^11^. Its major downstream effector, Rho-associated protein kinase (ROCK), plays a crucial role in cytoskeleton regulation by phosphorylating the myosin-binding subunit of myosin light chain (MLC) phosphatase, thereby inhibiting the myosin phosphatase activity and maintaining MLC in a contractile state^12^. This induces F-actin stress fibers formation and focal adhesions that are essential during cell migration^13^,^14^. ROCK also activates Lim kinase (LIMK) to inhibit cofilin, which prevents actin depolymerization, and further increases contractility by directly phosphorylating MLC^15^. Increased contractility can disrupt cell-cell adhesion and contributes to increased cell migration. There are evidences that E2 enhances endothelial cell migration through the Rho/ROCK pathway by acting through the ERs in endothelial cells^16^,^17^. However, the molecular mechanisms underlying the action of formononetin in ER, and the direct relationship with the action of ER and angiogenesis, are yet to be fully elucidated.

Endothelial cell proliferation and migration are essential processes in the development of angiogenesis which requires orchestrated movement of cells in particular directions to specific locations, closely involving the modification and spatial organization of actin cytoskeleton. Many phytoestrogens are shown to have pro-angiogenic effects, but whether phytoestrogens could induce effects on the regulation of cytoskeleton is unclear. In this study, we investigated the relationship of the pro-angiogenic actions of formononetin with ERα by performing molecular modeling and corresponding cell-based ERα transcriptional response reporter gene assay. The evaluation of formononetin-induced sprouting angiogenesis was also evaluated in zebrafish embryo *in vivo* and the formononetin-induced ROCK signaling in cytoskeleton reorganization and endothelial cell migration were evaluated in HUVEC.

## Results

### Identification of formononetin as a ligand at ERα by molecular docking analysis and cell-based GeneBLAzer enzymatic assay

**Figure 1:**
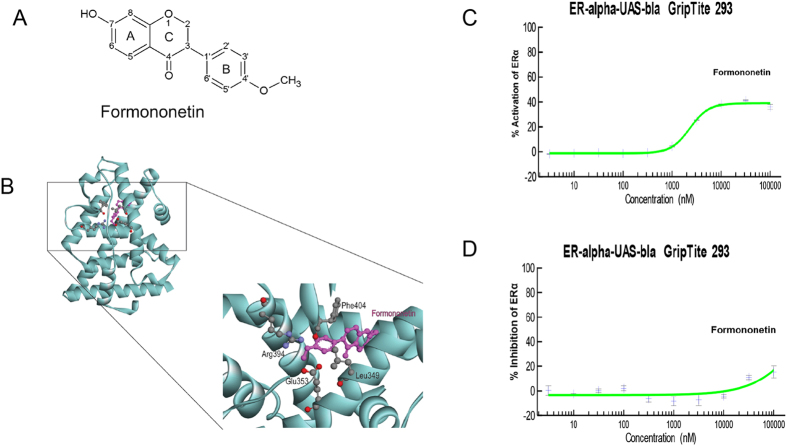
Formononetin exhibited direct binding and agonistic properties at ERα. (**A**) The molecular structure of formononetin. (**B**) Three-dimensional structural models of formononetin fitted into the ligand binding domain (LBD) of ERα. Formononetin was colored pink. Side chains in ERα LBD are colored by atom type (carbon, grey; oxygen, red; hydrogen, blue). (**C**) *In vitro* cell-based GeneBLAzer β-lactamase reporter-gene assay showed that formononetin induced the transactivation of ERα significantly. (**D**) Formononetin showed minimal inhibitory effects on the ERα transactivation induced by17-β-estradiol (E2). Results are presented as mean ± *SD* (*n* ≥ 2), *p* < 0.01.

It has been reported that formononetin possesses estrogenic activity. Therefore, we first studied the interaction between formononetin and the ligand binding pocket (LBD) of ERα by molecular docking. In general, ERα ligands form hydrogen bonds with Glu353 and Arg394 in the ERα LBD to promote structural stabilization. Figure 1A showed chemical structure of formononetin. Figure 1B illustrated that the methoxy group (–OCH_3_) at 4′-position of formononetin was able to form hydrogen bonds with the Glu 353 and Arg 394 of ERα LBD, and the carbonyl group (C=O) interacted with the Leu 349 of ERα LBD. We furthered investigated the actions of formononetin with cell-based GeneBLAzer nuclear receptor activation assay. Figure 1C showed that formononetin exhibited agonistic effect and induced the transactivation of ERα significantly. Figure 1D showed that formononetin in high concentration (100 μM) exhibited minimal inhibitory effects in 17-β-estradiol (E2)-induced ERα transactivation in the antagonistic experiment (Fig. 1D).

### Formononetin induced endothelial cell migration and stress fiber formation in HUVECs

**Figure 2:**
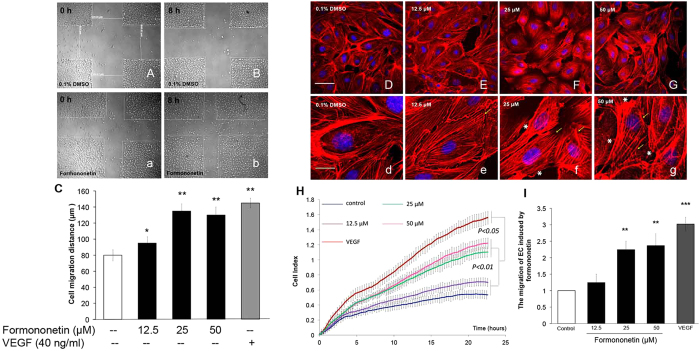
Formononetin induced cell migration and stress fiber formation in HUVECs. Formononetin enhanced the migration of HUVECs following scrape injury. (**A,B**) Serum-starved HUVEC monolayers were wounded (0 h) by micro-tips and washed with PBS, followed by addition of (**A,a**) 0.1% DMSO medium or (**B,b**) 25 μM formononetin for 8 h. (**C**) Statistical analysis of cell migration recorded by phase contrast microscopy following each scratch. The extent of migration was determined by averaging the mean length of the scraped area (gap width) of the migration pattern of endothelial cells. Formononetin also induced the rearrangement of actin cytoskeleton in HUVECs. Serum-starved HUVECs were treated with (**D,d**) 0.1% DMSO as control, and formononetin at (**E,e**) 12.5, (**F,f**) 25 and (**G,g**) 50 μM for 8 h. Cell nuclei were labeled with Hochest 33342 and F-actin was labeled with tetramethyl rhodamine isothiocyanate (TRITC)-phalloidin. Formononetin increased the formation of stress fibers that terminated at pointed edges (yellow arrowhead) and growth lamellipodia (white asterisk). White and yellow scale bars represent 50 and 20 μm, respectively. (**H**) HUVECs migration in response to different concentrations of formononetin was analyzed using the xCELLigence system. Real-time cell migrations of serum-starved HUVECs following the treatment of 12.5, 25 and 50 μM formononetin for 24 h were recorded. (**I**) Statistical analysis of the migration index of the xCELLigence system following formononetin treatments for 20 h. 40 ng/ml of VEGF was served as a positive control. Values are given as the increment of migration ± *SD* (*n* = 3), for three independent experiments. **p < *0.01*, **p < *0.001 vs*. control.

In angiogenesis, endothelial cells proliferation and migration contribute to the dissemination from pre-existing vessels to form new vessels. The effects of formononetin on endothelial cells proliferation and migration were investigated in human umbilical vein endothelial cells (HUVECs). Cell proliferation and cytotoxicity were first evaluated by using the XTT and LDH assays. It was observed that formononetin (12.5, 25, 50 or 100 μM for 48 h) did not induce HUVECs cell proliferation, and 100 μM formononetin caused cytotoxicity by inducing significant LDH release (Supplementary Fig. 1). We then further investigated the effect of formononetin on cell migration. It was observed that formononetin enhanced HUVECs migration following scrape injury (Fig. 2A–C). Quantitative analysis of the real-time migration of HUVECs in response to formononetin with the xCELLigence system showed that formononetin enhanced cell migration in a dose-dependent manner (Fig. 2H). More interestingly, it was found that formononetin induced actin cytoskeleton rearrangement in HUVECs and enhanced the formation of stress fibers (Fig. 2E,e–G,g). These stress fibers terminated at pointed edges and were typical morphological feature in migrating cells. These results indicated that formononetin stimulated HUVECs migration by inducing stress fiber formation.

### The role of ERα in formononetin-induced endothelial cell migration and stress fiber formation in HUVECs

**Figure 3:**
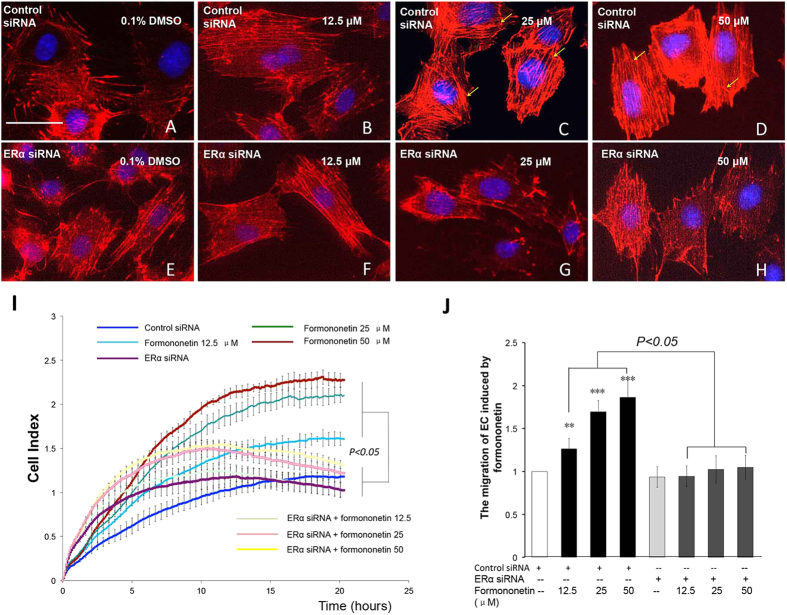
Inhibition of ERα expression by ERα siRNA abolished formononetin-induced stress fiber formation and cell migration in HUVECs. HUVECs were transfected with non-specific siRNA for 2 days followed by (**A**) 0.1% DMSO, (**B**) 12.5, (**C**) 25 and (**D**) 50 μM formononetin for 8 h to serve as negative control. HUVECs were transfected with ERα siRNA for 2 days followed by (**E**) 0.1% DMSO, (**F**) 12.5, (**G**) 25 and (**H**) 50 μM formononetin for 8 h. Cell nuclei were labeled with Hochest 33342 and F-actin was labeled with TRITC-phalloidin. Yellow arrowhead indicated stress fibers terminated at pointed edges. (**I**) Real-time cell migration of HUVECs transfected with ERα siRNA with or without formononetin treatments as detected by the xCELLigence system. HUVECs were transfected with non-specific siRNA or ERα siRNA for 2 days followed by 0.1% DMSO, 12.5, 25 or 50 μM formononetin for 24 h. (**G**) Statistical analysis of cell migration of HUVECs transfected with ERα siRNA following formononetin treatment for 20 h. Results are expressed as percentages of controls in mean ± *SD* (*n* = 3), **p < 0.01, ***p < *0.001 vs*. control.

**Figure 4:**
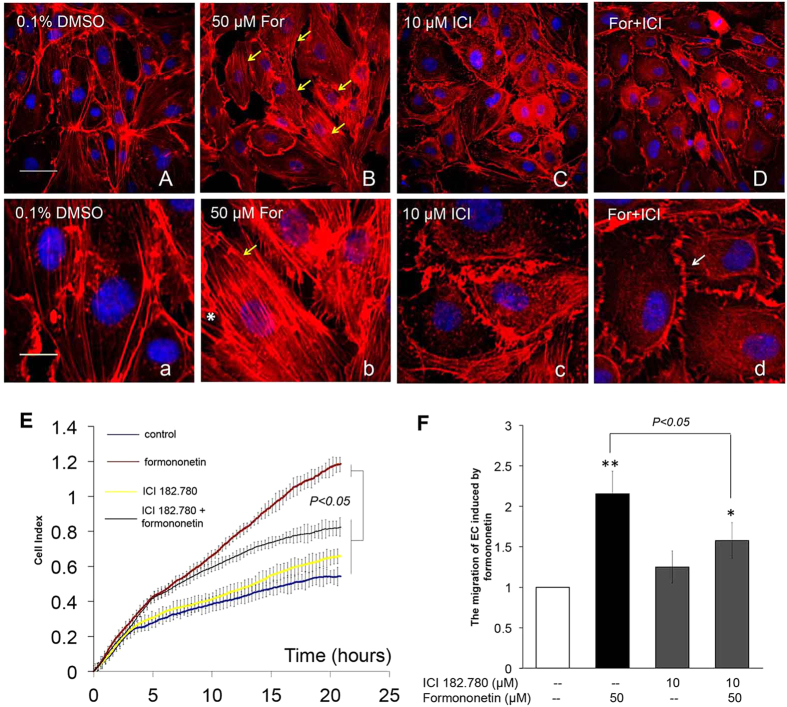
Blockade of ERα activity by ER antagonist ICI 182,780 abolished formononetin-induced stress fiber formation and cell migration in HUVECs. HUVECs were treated with (**A,a**) 0.1% DMSO for 8 h as negative control and (**B,b**) 50 μM formononetin for 8 h as positive control. For the experiments with ICI 182,780, HUVECs were first treated with ICI 182,780 (10 μM) for 2 h, followed by washout and the treatments of (**C,c**) 0.1% DMSO, and (**D,d**) 50 μM formononetin for 8 h. Cell nuclei were labeled with Hochest 33342 and F-actin was labeled with TRITC-phalloidin. Yellow arrowhead indicated stress fibers terminated at pointed edges. Cortical actin complexes (white arrowhead) were formed in the ICI 182,780 treatment group. (**E**) Real-time cell migration of HUVECs by using the xCELLigence system showed that ICI 182,780 inhibited formononetin-induced cell migration. HUVECs were treated with ICI 182,780 (10 μM) for 2 h, followed by washout and treatment with 0.1% DMSO or 50 μM formononetin for 24 h. (**F**) Statistical analysis of HUVECs cell migration following formononetin treatment for 20 h. Results are expressed as percentages of controls (means ± *SD*; *n* = 3), **p < 0.01, ***p < *0.001 vs*. control.

Since our molecular docking and enzymatic transactivation assay showed that formononetin exhibited direct binding and agonistic at ERα, we further investigated the role of ERα activity in the pro-angiogenic endothelial cell migration induced by formononetin in HUVECs. HUVECs were transfected with either non-specific siRNA to serve as negative control (Fig. 3A–D) or with siRNA against ERα mRNA (Fig. 3E–H). It was observed that the inhibition of ERα expression in HUVECs significantly prevented the increased stress fiber formation induced by formononetin. Figure 3I,G showed that the concurrent cell migrations of HUVECs induced by formononetin treatments were also significantly inhibited by silencing ERα with siRNA. Similarly, the estrogen receptor antagonist ICI 182,780 significantly reduced the formononetin-induced stress fiber formation and cell migration (Fig. 4).

### Formononetin induced stress fiber formation and cell migration in HUVECs through ROCK and MMP2/9 signaling pathways

**Figure 5:**
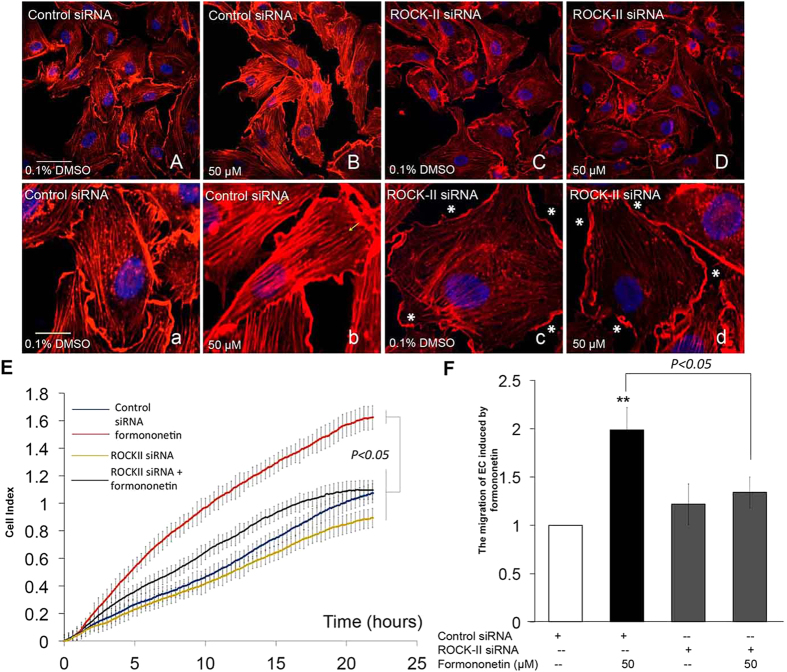
Inhibition of ROCK-II expression by ROCK-II siRNA abolished formononetin-induced stress fiber formation and cell migration in HUVECs. HUVECs transfected with non-specific siRNA were treated with (**A,a**) 0.1% DMSO or (**B,b**) 50 μM formononetin for 8 h to serve as negative and positive control, respectively. Cell nuclei were labeled with Hochest 33342 and F-actin was labeled with TRITC-phalloidin. Yellow arrowhead indicated stress fibers terminated at pointed edges. ROCK-II expression was inhibited in HUVECs by using ROCK-II siRNA and the cells were treated with (**C,c**) 0.1% DMSO or (**D,d**) 50 μM formononetin for 8 h. White asterisk indicated cortical actin complexes formed in HUVECs transfected with ROCK-II siRNA. White and yellow scale bars represent 50 μm and 20 μm respectively. (**E**) Real-time cell migration of HUVECs transfected with ROCK-II siRNA with or without formononetin treatments as detected by the xCELLigence system. HUVECs were transfected with ROCK-II siRNA for 2 days, followed by 0.1% DMSO or 50 μM formononetin for 24 h. (**F**) Statistical analysis of cell migration index of xCELLigence system following formononetin treatment for 20 h. Values are presented as the increment of migration ± *SD* (*n* = 3), for three independent experiments. **p < *0.01 vs*. control.

**Figure 6:**
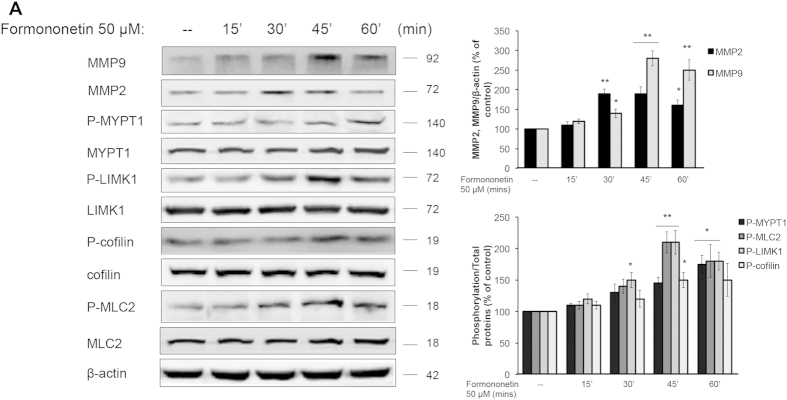
Formononetin induced the activation of ROCK and MMP2/9 signaling pathways in HUVECs. HUVECs were treated with 50 μM formononetin for 15, 30, 45, 60 min. Cells were subsequently harvested and extracted for western blot analysis. (**A**) The protein expression of the ROCK signal pathway and MMP2/9 were determined by western blotting. β-actin was used as a loading control. (**B,C**) Results are expressed as a percentage of controls (mean ± *SD*; *n* = 3), *p < 0.05, **p < *0.01 vs*. control.

**Figure 7:**
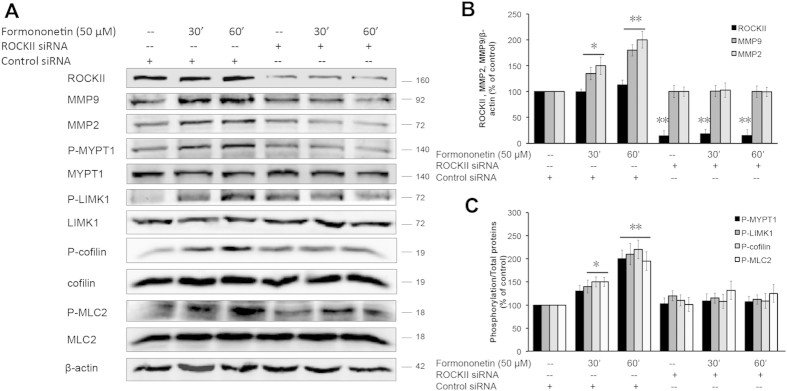
Inhibition of ROCK-II expression by ROCK-II siRNA abolished the activation of ROCK and MMP2/9 signaling pathways in HUVECs. (**A**) HUVECs were transfected with either non-specific siRNA as control or specific ROCK-II siRNA for 2 days followed by 50 μM formononetin treatment for 30 or 60 min. The protein levels of ROCK-II, MMP2, MMP9, P-MYPT1, P-LIMK1, P-cofilin and P-MLC2 were analyzed by using Western blotting. (**B**) Statistical analysis of the protein levels of MMP2 and MMP9 in HUVECs transfected with ROCK-II siRNA in the presence of 50 μM formononetin. (**C**) Statistical analysis of the protein levels of P-MYPT1, P-LIMK1, P-cofilin and P-MLC2 in HUVECs transfected with ROCK-II siRNA in the presence of 50 μM formononetin. Results are expressed as a percentage of control (means ± *SD*, *n* = 3), *p < 0.05, **p < *0.01 vs*. control.

We further investigated the underlying mechanism of the formononetin-induced actin cytoskeleton rearrangement in HUVECs. Previous studies have demonstrated that the activation of ROCK signaling pathway is closely related to the regulation of stress fiber formation and cell migration in endothelial cells^13^,^14^. Figure 5B,b showed that HUVECs transfected with non-specific siRNA when treated with formononetin (50 μM), actin stress fiber formation was increased significantly and the fibers were arranged longitudinally through the major axis of the endothelial cells. When HUVECs were transfected with ROCK-II siRNA, there was a reduction in actin stress fiber as well as a rapid modification of the stress fiber spatial organization with a progressive localization of actin towards the edge of the cell membrane to form cortical actin complexes (Fig. 5C,c). More interestingly, the inhibition of ROCK-II expression by siRNA abolished the actions of formononetin to increase stress fiber formation (Fig. 5D,d) as well as cell migration (Fig. 5E,F). Moreover, the cell migration of wounded HUVECs induced by formononetin treatment following scrape injury was completely abolished by the transfections of either ERα siRNA or ROCK-II siRNA (Supplementary Fig. 2). The effects of formononetin on the regulations of the downstream effectors of ROCK activation were further evaluated by using western blotting. The downstream substrates of ROCK proteins including MMP2/9 (matrix metalloproteinase 2/9), MYPT1 (myosin phosphatase target subunit 1), LIMK1 (Lim kinase 1), MLC2 (myosin light chain 2) and cofilin have been shown to regulate cell migration. Our data showed that formononetin increased the expressions of MMP2 and MMP9 in HUVECs significantly in a time-dependent manner (Fig. 6A,B). Formononetin also concurrently increased the phosphorylation of MYPT1, MLC2, LIMK1 and cofilin as shown in Fig. 6A,C. These effects induced by formononetin were abolished by the transfection of ROCK-II siRNA in HUVECs. Figure 7 showed that the transfection of ROCK-II siRNA resulted in marked reduction in the expression of ROCK-II protein along with similar decrease in the phosphorylation of MYPT1, MLC2, LIMK1, cofilin and the expression of MPP2 and MPP9 in formononetin treated HUVECs (Fig. 7).

### The role of ERα in the activation of ROCK and MMP2/9 pathways in formononetin-induced stress fiber formation and cell migration in HUVECs

**Figure 8:**
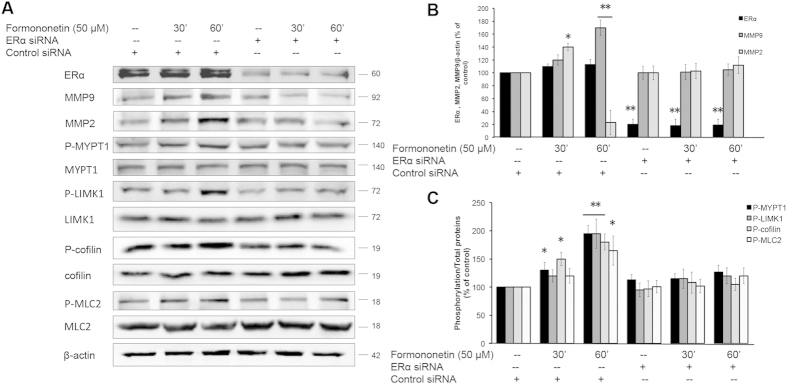
Formononetin induced the activations of ROCK and MMP2/9 pathways via ERα. (**A**) HUVECs were transfected with either non-specific siRNA as control of specific ERα siRNA for 2 days followed by 50 μM formononetin treatment for 30 or 60 min. The protein levels of ERα, MMP2, MMP9, P-MYPT1, P-LIMK1, P-cofilin and P-MLC2 were analyzed by using Western blotting. (**B**) Statistical analysis of the protein levels of MMP2 and MMP9 in HUVECs transfected with ERα siRNA in the presence of 50 μM formononetin. (**C**) Statistical analysis of the protein levels of P-MYPT1, P-LIMK1, P-cofilin and P-MLC2 in HUVECs transfected with ERα siRNA in the presence of 50 μM formononetin. Results are expressed as percentages of controls (mean ± *SD*; *n* = 3), *p < 0.05, **p < *0.01 vs*. control.

**Figure 9:**
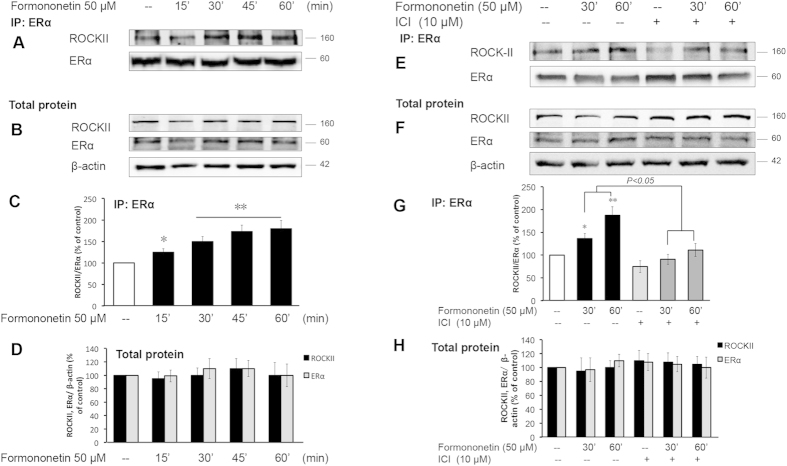
Inhibition of ER activation by ICI 182,780 abolished the interaction between ERα and ROCK-II induced by formononetin. (**A–D**) HUVECs were treated with 0.1% DMSO or 50 μM formononetin for 15, 30, 45 and 60 min. (**E–H**) HUVECs were pretreated with 10 μM ICI 182,780 for 2 h, followed by washout and treatment with 0.1% DMSO or 50 μM formononetin for 30 and 60 min. HUVECs protein extracts were immunoprecipitated with anti-ERα antibody and the immunoprecipitations (IPs) were assayed for co-immunoprecipitation of ROCK-II with Western blotting. HUVECs total protein was directly assayed with western blotting analysis as controls. Results are expressed as percentage of control in mean ± (*n* = 3), *p < 0.05, **p < *0.01 vs*. control.

The role of ERα in the activation of ROCK and MMP2/9 pathways by formononetin was further evaluated by using ERα siRNA. Figure 8 showed that HUVECs transfected with ERα siRNA resulted in a marked reduction in ERα expression along with marked reductions in the protein levels of MMP2 and MMP9, and the phosphorylation of MYPT1, LIMK1, cofilin and MLC2 upon exposure to formononetin. To further characterize the signaling partnership between ERα and ROCK, co-immunoprecipitation assays were performed. It was observed that in the presence of formononetin, there was significant increased in the interaction between ERα and ROCK-II (Fig. 9A–D). In order to dissect the functional relevance of the ERα/ROCK-II interaction, we further investigated if the ERα-associated ROCK-II is functionally activated by using the ER antagonist ICI 182,780. It was observed that the treatment of ICI 182,780 disrupted the interaction between ERα/ROCK-II induced by formononetin (Fig. 9E–H). These results suggested that formononetin induced direct association of ERα with ROCK-II that further led to the formation of an activated multi-protein complex.

### Formononetin induced angiogenic sprouting in subintestinal vessels (SIVs) in zebrafish *in vivo*

**Figure 10:**
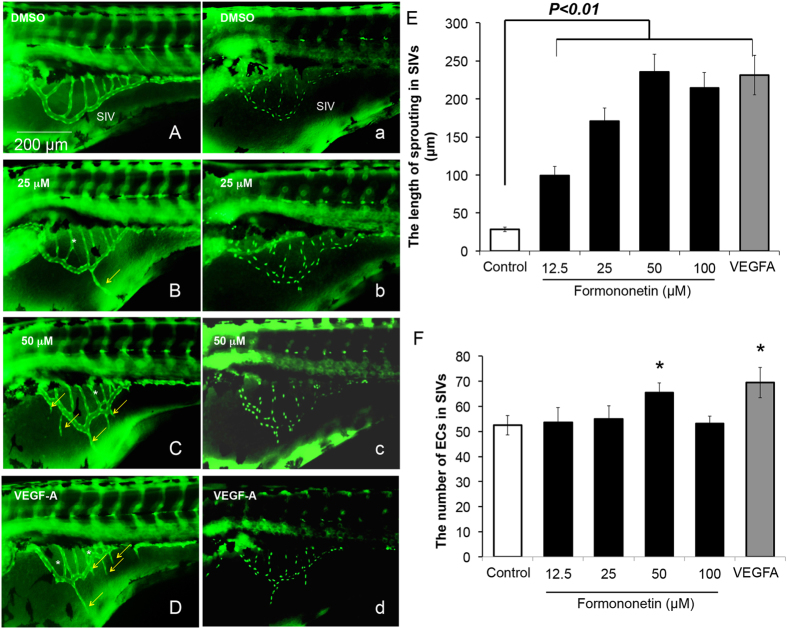
The effects of formononetin on angiogenic sprouting in the SIVs of *Tg (fli1: EGFP)y1* and *Tg (fli1: nEGFP)y7* zebrafish embryos. (**A,a**) *Tg (fli1: EGFP)y1* and *Tg (fli1: nEGFP)y7* zebrafish embryos (48 hpf) were treated with 0.1% DMSO for 24 h to serve as negative control. The SIVs of zebrafish embryos at 72 hpf developed into a smooth basket-like structure. Zebrafish embryos (48 hpf) treated with formononetin for 24 h at (**B,b**) 25 μM or (**C,c**) 50 μM showed that there was an increase of sprouting in the subintestinal vessels (SIVs) baskets stretching into the posterior yolk extension. (**D,d**) Zebrafish embryos were injected with zebrafish VEGF-A (ZF-VEGF-A) (1 ng/embryo) at 1 hpf and incubated in embryo medium until 72 hpf to serve as positive control. Yellow arrows indicated sprouting vessel formation; white asterisks indicated intersection branch formation. Statistical analysis showed that formononetin increased (**E**) the length of vascular sprouting in the SIVs and (**F**) the number of endothelial cells in the SIVs in a dose-dependent manner. The number of endothelial cells was measured using Image J software package. Data are plotted as mean ± *SD*, (*n* > 3), *p < 0.05, **p < 0.01 *vs*. control.

In order to determine if formononetin exerted pro-angiogenic effects *in vivo*, we employed the transgenic zebrafish models *Tg (fli1: EGFP)y1* and *Tg (fli1: nEGFP)y7*. *Tg (fli1: EGFP)y1* zebrafish were engineered to express green fluorescent protein (GFP) in the entire vasculature and *Tg (fli1: nEGFP)y7* harbored nuclear-localized GFP expression in the endothelial cells permitting real time *in vivo* analysis of individual endothelial cells. Figure 10A showed that under normal condition, zebrafish embryos developed a smooth, basket-like structure in the region of the subintestinal vessels (SIVs) at 72 hour post fertilization (hpf). The treatments of formononetin at 25 μM (Fig. 10B,b) or 50 μM (Fig. 10C,c) increased the sprouting in the SIVs in a dose-dependent manner. The increase in sprouts formation induced by formononetin was similar to the effect of zebrafish VEGF-A (ZF-VEGF-A) (1 ng/embryo) (Fig. 10D,d). We further evaluated the phenotype and the behavior of individual endothelial cells to determine if the pro-angiogenic effects of formononetin were caused by endothelial cell proliferation and migration. Quantitative analysis indicated that formononetin (50 μM) significantly induced an increase in the length of sprouting vessels (Fig. 10E) as well as a slightly increase in the endothelial cell populations throughout the region of the SIVs (Fig. 10F), similar to the effects induced by ZF-VEGF-A injection.

**Figure 11:**
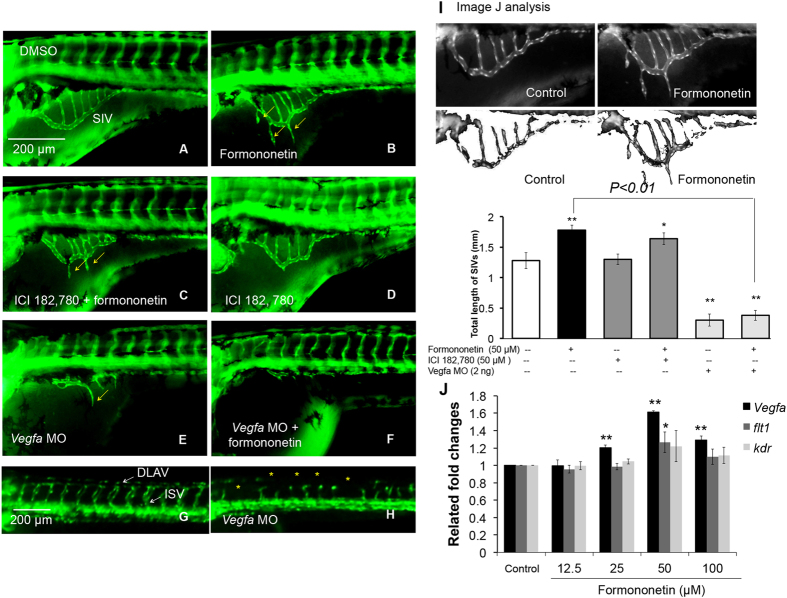
The effects of ICI 182,780 (ER antagonist) and *vegfa* morpholino on formononetin-induced angiogenesis in zebrafish embryos *in vivo*. *Tg (fli1: EGFP)y1* zebrafish embryos (48 hpf) were treated with (**A**) 0.1% DMSO (negative control), (**B**) formononetin (50 μM) (positive control), (**C)** co-treatment of ICI 182,780 (50 μM) and formononetin (50 μM), (**D**) ICI 182, 780 (50 μM) alone. All treatments were for 24 h. *Tg (fli1: EGFP)y1* zebrafish embryos (1 hpf) were injected with (**E**) *Vegfa* MO (2 ng) alone and (**F**) *Vegfa* MO (2 ng) injection + formononetin treatment (50 μM, at 48 hpf for 24 h) and were observed at 72 hpf. (**G,H**) Control images of zebrafish embryos at 1–4 cells stage and 24 hpf after *Vegfa* MO injection. (**I**) Data were analyzed by using the Image J software package. Quantitative analysis indicated the total length of SIVs for each group. (**J**) Evaluation of gene expressions in formononetin-treated zebrafish embryos by using real-time PCR. Data are plotted as means ± *SD* from three individual experiments. *p < *0.05*, **p < *0.01 vs*. control group.

The bars chart in Fig. 11J represent the gene expression of angiogenesis factors after treatment with 50 μM formononetin for 6 h. There was a significant increase of the mRNA expressions of VEGF-A (1.6-fold at 50 μM; *p* < *0.01*) and Flt1 (or VEGFR1) (1.25-fold at 50 μM; *p* < *0.05*) but not Kdr (or VEGFR2) (1.18-fold at 50 μM; *p* *>* *0.05*) compared to the control.

### Formononetin induced angiogenic sproutings in the SIVs of zebrafish embryos *in vivo* through multiple signaling pathways with ROCK contributing a key role

We further investigated the role of estrogen receptors (ER) in the pro-angiogenic sproutings induced by formononetin in zebrafish embryos by using the ER antagonist ICI 182,780. Figure 11A showed that under normal condition. Figure 11B showed the treatment of formononetin at 50 μM increased the sprouting in the SIVs. Figure 11C showed the treatment of ICI 182,780 no significantly changed the morphology of SIVs. Figure 11D showed that in the presence of ICI 182,780 (50 μM), the formononetin-induced sproutings in the SIVs were partially inhibited. The statistical analysis in Fig. 11I showed that this inhibition induced by ICI 182,780 was modest but significant. In addition, it was also observed that ICI 182,780 was unable to reduce the up-regulation of VEGF-A expression stimulated by formononetin treatment in zebrafish embryos (data not shown). Previous studies have demonstrated that VEGF-A morphant zebrafish embryos failed to develop complete vasculature with complete absence of the intersegmental and axial vasculature^18^. In the present study, we also observed that VEGF-A morphant embryos (72 hpf) failed to develop the SIVs (subintestinal vessels) (Fig. 11E,H), and that the angiogenic sproutings induced by formononetin were abolished by the injection of VEGF-A morpholino (VEGF-A MO) (Fig. 11F).

**Figure 12:**
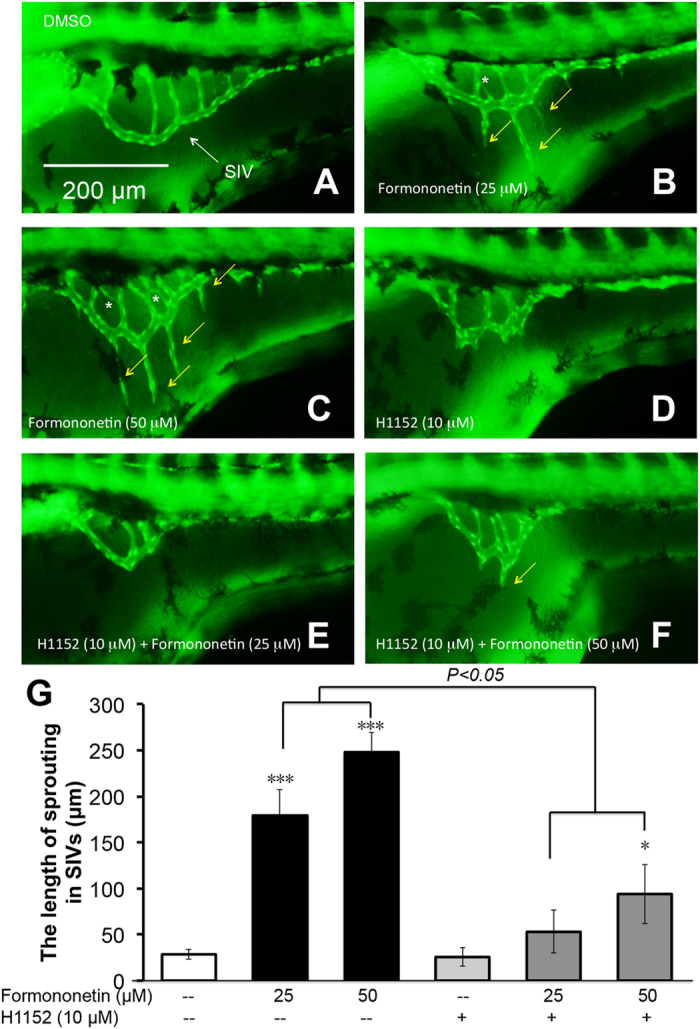
The effects of ROCK inhibitor in formononetin-induced angiogenesis in zebrafish embryos *in vivo*. *Tg (fli1: EGFP)y1* zebrafish embryos (48 hpf) were treated with (**A**) 0.1% DMSO (negative control), (**B**) formononetin (25 μM), (**C**) formononetin (50 μM), (**D**) H1152 (10 μM, ROCK inhibitor) alone, (**E**) co-treatment of H1152 (10 μM) +formononetin (25 μM), (**F**) co-treatment of H1152 (10 μM) +formononetin (50 μM). All treatments were for 24 h. (**G**) Quantitative analysis indicated the length of sprouting in SIVs for each group. Data are plotted as means ± *SD*, from three individual experiments. *p < *0.05*, ***p < *0.001 vs*. control group.

We further investigated the role of the ROCK pathway in formononetin-induced sproutings *in vivo* in zebrafish by using the ROCK inhibitior H1152. In agreement with the results in HUVECs, H1152 abolished the sproutings induced by formononetin significantly (Fig. 12D–F). These results suggested that formononetin induced angiogenesis in zebrafish embryos via multiple pathways and various steps in angiogenesis (e.g. cell proliferation and migration).

## Discussion

Angiogenesis is not only a prerequisite for physiological tissue development and regeneration, but also an integral part of chronic disease pathogenesis. Therefore, it is not surprising that therapies targeting angiogenesis have been sought from both perspectives. Pro-angiogenic therapies have been investigated for treating ischemic heart disease, wound healing, and other disease settings with an impaired blood supply^19^,^20^. On the contrary, in cancer, diabetic retinopathy, rheumatoid arthritis and other diseases where neovascularization is a problem, efforts have been made to develop therapies that inhibit angiogenesis^21^,^22^. In the present study, we investigated the underlying mechanisms of the angiogenic effects induced by formononetin. Formononetin is an isoflavone that has been shown to display estrogenic properties^23^,^24^. Formononetin is also an active ingredient in *Radix Astragali*, a Chinese medical herb that promotes hind limb ischemia-induced wound healing in diabetic rats by promoting angiogenesis^25^,^26^,^27^. Here we showed for the first time that formononetin displayed direct binding to the LBD (ligand binding domain) of ERα (estrogen receptor alpha) by using molecular docking. Moreover, the data from the cell-based GeneBLAzer assays which evaluated the transactivation of ERα showed that formononetin demonstrated agonistic activity at ERα. It is now known that ERα are expressed in endothelial cells, and ERα agonists could induce endothelial cell proliferation and migration, and mediate angiogenesis^7^,^28^.

It has been reported that formononetin promoted tissue healing in early fracture through stimulating angiogenesis by up-regulating growth factors including VEGF and Flk-1 in a rat fracture model^5^. In another report, formononetin was observed to induce endothelial cell proliferation and migration of wounded HUVECs by significantly increasing growth factors^6^. In line with this, our *in vivo* data in zebrafish embryos showed that the gene expressions of VEGF-A and Flt1 were significantly up-regulated by formononetin treatment, and the injection of *vegfa* morpholino disrupted the normal vasculature development in zebrafish embryos and the addition of formononetin was unable to produce any angiogenesis in the VEGF-A morphant. These results indicated that formononetin promoted angiogenesis to induce sprouting formation in zebrafish. Moreover, the proangiogenesis activities of formononetin are also through VEGF signaling pathway. However, angiogenesis is a multi steps process with endothelial cell proliferation, migration and capillary network formation as the fundamental procedures. Therefore, we further elucidated the underlying mechanisms of formononetin-induced pro-angiogenic effects on the endothelial cells by using HUVECs. In the present study, we observed for the first time that formononetin induced endothelial cell migrations and dramatic actin stress fiber formation and spatial modification of the actin cytoskeleton in endothelial cells. Moreover, formononetin did not induce any significant increase in endothelial proliferation. Formononetin induced the rearrangement of actin cytoskeleton into the migratory phenotype (stress fibers terminated at pointed edges and concentrated at growth lamellipodia). Further investigation by using ICI 182,780 (estrogen receptors antagonist) and anti-ERα siRNA showed that ERα activation contributed significantly to the formononetin-induced cell migration and stress fiber formation. These effects were associated with an increase in matrix metalloproteinase MMP2/9 expressions and the activation of the ROCK (Rho-associated protein kinase) signaling pathway. It has been reported that MMP2 and MMP9 are predominately expressed in the endothelial cells and are directly involved in endothelial cell migration and vascular remodeling during angiogenesis^29^. ROCK is a major downstream effector of RhoA, and contributes to stress fiber formation by inactivating myosin phosphatase and by phosphorylating myosin light chain. ROCK also activates Lim kinase (LIMK) to phosphorylate and thereby inhibiting cofilin to prevents actin depolymerization. Increased actin depolymerization and myosin contractility resulted in F-actin stress fibers formation and tension that play essential roles in cell migration^13^,^14^. Results from this study showed that formononetin increased protein expression of MMP2/9 and ROCK-II, and enhanced the phosphorylation of its major downstream effectors and substrates including MYPT1 (myosin phosphatase target subunit 1), LIMK1 (Lim kinase 1), MLC2 (myosin light chain 2) and cofilin. The increased in MMP2/9 and the enhanced phosphorylation of MYPT1, LIMK1, MLC2 and cofilin were all inhibited by the anti-ROCK-II siRNA. The results provided strong evidence that ROCK signaling pathway is a major mechanism underlying the pro-angiogenic cell migration induced by formononetin. In addition, in agreement with the results above that anti-ERα siRNA inhibited formononetin-induced stress fiber formation and cell migration, anti-ERα siRNA or ICI 182,780 also inhibited the formononetin-induced MMP2/9 expression and ROCK signaling activation. Indeed, “non-genomic” effects of estrogens has been reported which acts on endothelial cell to modify actin cytoskeleton arrangement and endothelial cell migration via the activation of surface ERα, recruiting RhoA/ROCK-II/moesin cascade^16^,^30^. We further provided more evidence of the interaction between ERα and ROCK-II by using co-immunoprecipitation, and the ERα/ROCK-II interaction was disrupted by ICI 182,780. To further investigate formononetin-induced the activation of ROCK signaling pathway may also be related the interactions of formononetin to factors other than ERα, we then applied docking analysis to simulate the interaction between formononetin and upstream factors of ROCK signaling, such as VEGFR2, TGF-beta receptor 1, or RhoA (Supplementary Fig. 3). Through the docking analysis, formononetin strongly bound to LBD of ERα, very weakly bound the kinase activation site of VEGFR2 and TGF-beta receptor 1, but did not bind to RhoA (Supplementary Table 1). These results further confirmed that formononetin induced the activation of ROCK signaling pathway via the interaction with ERα. Interestingly, previous study showed that formononetin in high concentrations (>50 μM) acted as a strong pro-apoptotic agent inducing antiangiogenesis in cancer cell lines^31^,^32^. In line with previous study, we found that formononetin in high concentrations (>50 μM) could also induce cell death in HUVECs (Supplementary Fig. 1). Overall, according to previous study and our current results, we assume that formononetin in low concentrations (<50 μM) have proangiogenesis effects while in high concentrations (>50 μM) display anti-angiogenesis effects. These results support formononetin can be used in the treatment of insufficient and sufficient angiogenesis diseases such as ischemic heart disease, wound healing and metastatic cancers.

**Figure 13:**
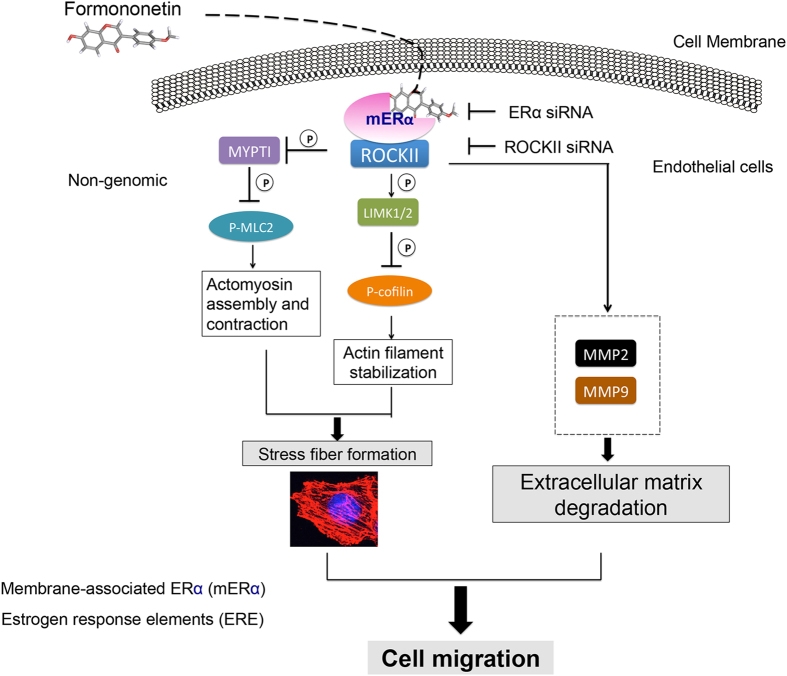
Formononetin induced pro-angiogenic actin stress fiber formation and cell migration in endothelial cells involving ERα/ROCKII signaling pathways as major regulators.

Taken together, these results lead us to postulate that formononetin induced endothelial cell migration and stress fiber formation and promoted angiogenesis through an ERα-enhanced ROCK pathway by forming an ERα/ROCK-II activated complex (Fig. 13). These results expand our knowledge pertaining to the basis of the effects of phytoestrogens in angiogenesis, including the regulation of ERα and ROCK interaction relevant to actin assembly and cell migration. This new concept might provide support for the pro-angiogenic activities of formononetin and other phytoestrogens during the treatment of conditions related to vascular insufficiency, such as wound healing and diabetic vascular complications.

## Methods

### Ethical Statement

The experiments in this manuscript comply with the current laws of Macau. All the animal experiments in this manuscript were conducted according to the ethical guidelines of the Institute of Chinese Medical Sciences (ICMS), University of Macau. The protocol was approved by the Institute of Chinese Medical Sciences—Animal Ethics Committee (ICMS-AEC; Permit Number: 20120601). All experiments were performed in accordance with relevant guidelines and regulations.

### Chemicals and reagents

Human Umbilical Vein Endothelial Cells (HUVEC) were obtained from Thermo Fisher Scientific Inc. (NYSE: TMO), maintained in Vascular Cell Basal Medium ATCC (Manassas, VA), and used prior to passage 7. Fetal bovine serum (FBS), phosphate-buffered saline (PBS), penicillin-streptomycin (PS) and 0.25% (w/v) trypsin/1 mM EDTA were all purchased from Invitrogen (Carlsbad, CA, USA). Endothelial cell growth supplement (ECGS), heparin and gelatin were all supplied by Sigma (St Louis, MO). Vascular endothelial growth factors (VEGF) were obtained from R&D Systems (Minneapolis, MN). Anti-MMP2/9 antibody, Anti-ROCK-II antibody, anti-MYPT1 antibody, Anti-LIMK1 antibody, Anti-cofilin antibody, Anti-MLC2 antibody, Anti-β-actin antibody and goat anti-rabbit IgG HRP-conjugated antibody were all purchased from Cell Signaling Technology (Berverly, MA). Anti-ERα Antibody was purchased from Santa Cruz Biotechnology, Inc. Dimethyl sulfoxide (DMSO) was acquired from SIGMA. Formononetin was obtained from the Chinese National Institute for Control of Pharmaceutical and Biological Products (Beijing, China) and was dissolved in DMSO to form a 50 mM solution.

### Molecular docking study

Molecular docking analysis was performed to investigate the binding of formononetin to human estrogen receptor alpha (ERα) (PDB code 2QZO) which were obtained from the Protein Data Bank. The software AutoDcok Vina ver. 4.2 was used for all dockings^33^,^34^. A Lamarckian genetic algorithm (LGA) was used to search for conformations using the following docking parameters: a population size of 150 individuals; a maximum number of generations and energy evaluations of 27000 and 25 million, respectively; and 50 docking runs and random initial positions and conformations. The binding energy of each conformation was calculated using the AMBER force field, and a root mean squared deviation (R.M.S.D) tolerance of 0.7 Å was used to cluster the conformations using the AutoDock Tools software package. The lowest energy, and highest cluster conformations, of two molecules were further analyzed and compared using the Discovery Studio software package (Accelrys).

### Zebrafish embryo preparation and drug treatments

All animal experiments were undertaken following the approved protocol of Institute of Chinese Medical Sciences of the University of Macau. Zebrafish embryo preparation proceeded as described in our previous paper^3^. Transgenic *Tg (fli1: EGFP)y1* and *Tg (fli1: nEGFP)y7* zebrafish were maintained separately under a 14 h light/10 h dark cycle and standard conditions. Embryos were generated by pairwise mating (of fish between 3 and 12 months of age) and incubated at 28.5 °C in embryo medium. Only embryos aged 48 h post-fertilization (hpf) were used in the experiments. For morphological analysis, 12.5, 25, 50 and 100 μM formononetin were dissolved directly in 2 ml MilliQ water, in a twelve-well microplate, with 15 individual embryos in each treatment group. The vehicle control group was treated under the same conditions, but drug was replaced with 0.1% DMSO. All groups were incubated at 28 °C for 24 h.

### Morphological observation of zebrafish

At 96 hpf, embryos taken from microplates were observed for viability and gross morphological changes under a fluorescence microscope (Olympus IX81 Motorized Inverted Microscope, Japan) equipped with a digital camera (DP controller, Soft Imaging System, Olympus). For the assessment of vascular changes, images were analyzed using Axiovision 4.2 and Adobe Photoshop 7.0, to quantify vascular changes. The quantitative analysis of the SIVs of *Tg (fli1: EGFP)y1* zebrafish embryo were chosen for the measurement of total vessels or sprouting vessels length in SIVs, using the AxiovisionLE software package (ver. 4.1) and Image J.

### Morpholino and VEGF-A injections

Morpholino antisense oligonucleotides were purchased from Gene-Tools, LLC (Philomath, OR). A 1 nl volume of 2 mM morpholino or control morpholino (Gene-Tools, Philomath, OR), was injected into transgenic *Tg (fli-1: EGFP)y1* embryos (approximately 2 ng *Vegfa* MO) at the 1- to 4-cell stage. The sequence of the injected translation-blocking target morpholino was as follows: *Vegfa* (NM_001044855.2) 5′-GGAGCACGCGAACAGCAAAGTTCAT-3′. Zebrafish VEGF-A (1247—ZV- 010, R&D Systems) injection has been described elsewhere^35^. Briefly, we injected approximately 10–15 nl of solution (approximately 1.0–1.5 ng VEGF-A, or PBS as control) into either AB or transgenic *Tg (fli-1: EGFP)y1* and *Tg (fli-1:n EGFP)y7* embryo yolks at the 1- to 4-cell stage. All injected embryos were photographed live under a fluorescence microscope (Olympus IX81 Motorized Inverted Microscope, Japan) using an Olympus DP controller, and the Soft Imaging System software package.

### Cell-based ER transcriptional response by GeneBLAzer nuclear receptor activation assay

Assays were performed by Invitrogen (USA) as described in the literature^3^. GeneBLAzer β-lactamase reporter-gene assays were performed to measure the agonistic or antagonistic activities of formononetin at ER, the methods for which were described in our previous paper^3^. For the ER agonist activity assay, 17-β-estratiol served as the control agonist. For the antagonist activity assay, 4-hydroxytamoxifen (starting concentration of 100 nM) for ERα served as the control antagonist. 80% effective concentration (EC_80_) of 17-β-estradiol (E2) was determined and used as the baseline measure of activation or 0% inhibition.

### Quantitative real time PCR (qRT-PCR)

Total RNA extraction, reverse transcription, and real-time PCR of zebrafish embryos, at 48 hpf, were treated with 12.5, 25 and 50 μM formononetin for 6 h. Total RNA was extracted from 30 zebrafish embryos of each treatment group using the RNeasy Mini Kit (Qiagen, USA) in accordance with the manufacturer’s instructions. RNA was reverse-transcribed to single-strand cDNA using the SuperScriptTM III First- Strand Synthesis System for RT-PCR (InvitrogenTM, USA), followed by real-time PCR using TaqManH Universal PCR Master Mix and 250 nM custom TaqMan primers for zebrafish *Kdr*, *Flt1*, and *VEGF-A* (Applied Biosystems, USA), in the ABI 7500 Real-Time PCR System (Applied Biosystems). The expression of *Kdr*, *Flt1*, and *VEGF-A* mRNA was normalized to the amount of bactin1, using the relative quantification method described by the manufacturer. The zebrafish beta-actin1 primers were 5′-CATCGGCAATGAGCGTTTCC-3′ (F) and 5′-CAAGATTCCATACCCAGGAAGG A-3′(R) (Applied Biosystems, USA). The zebrafish *Kdr* primers were 5′-CAAGTAACTCGTTTTCTCAACCTAAGC-3′ (F) and 5′-GGTCTGCTACACAAC GCATTATAAC-3′(R) (Applied Biosystems, USA). The zebrafish *Flt1* primers were 5′-AACTCACAGACCAGTGAACAAGATC-3′ (F) and 5′-GCCCTGTAACGTG TGCACTAAA-3′(R) (Applied Biosystems, USA). The zebrafish *VEGF-A* primers were 5′-GATGTGATTCCCTTCATGGATGTGT-3′ (F) and 5′-GGATACTCC TGGATGATGTCTACCA-3′ (R) (Applied Biosystems, USA). Data analyses were obtained from at least three independent experiments.

### HUVEC culture

Human Umbilical Vein Endothelial Cells (HUVEC) were obtained from Thermo Fisher Scientific Inc. (NYSE: TMO), and maintained in Vascular Cell Basal Medium ATCC (Manassas, VA) at 37 °C in a humidified atmosphere of 5% CO_2_. Tissue culture flasks, 96-well plates and 6- well plates were pre-coated with 2 μg/cm^2^ fibronectin. Cultures were then starved with low-serum medium (containing 0.5% CS-FBS) overnight in cell viability, toxicity and migration assays. All assays were conducted using low cell passage cells (2–6 passages).

### HUVEC viability by XTT assay

HUVECs were trypsinized and seeded at 10^4^ cells/well, in 96-well gelatin coated plates. After 24 h, complete medium was removed and renewed with hormone-free low serum (0.5% CS-FBS) medium. Samples were then incubated for 24 h, to starve HUVECs and achieve a quiescent state. Following these pre-incubations, different concentrations (12.5 μM–100 μM) of formononetin medium were replaced. Cells receiving DMSO (0.1%) served as vehicle controls, and were equivalent to no treatment. After 24 or 48 h, cell proliferation was assessed by XTT for 4 h. The spectrophotometrical absorbance of each well was measured by a Multilabel counter (Perkin Elmer, Singapore). The wavelength used to measure absorbance of the formazan product was 490 nm. The reference wavelength was 690 nm. Cell viability data were expressed as a percentage of calculated cell viability.

### Lactate dehydrogenase (LDH) assay

HUVEC were grown to 80% confluency on 96 wells plate, and were then treated with 12.5, 25, 50 and 100 μM formononetin for 24 h. Cellular toxicity was determined by measuring the activity of LDH released into the incubation medium. Released LDH activity was determined by the cytotoxicity detection kit according to the manufacturer’s instructions. Absorbance was measured using a microplate reader at 490 nm.

### Gene silencing with RNA interference

The synthetic siRNA targeting ERα (sc-29305, Santa Cruz Biotechnology) and ROCK-II siRNA (h) (sc-29474, Santa Cruz Biotechnology) were used at the final concentration of 100 nM to silence ERα and ROCK-II, with Lipofectamine^®^ LTX & Plus Reagent (15338-100, Life Technologies Corporation) according to the manufacturer’s instructions. Endothelial cells were incubated in complete medium for 48 h following siRNA transfection. The efficacy of gene silencing was checked with western blot analysis and discovered to be optimal at 48 h.

### Cytoskeleton staining

HUVEC were seeded onto fibronectin-pretreated sterile coverslips placed into 96-well plates, and exposed to formononetin for 8 h. Cells were then washed, fixed, permeabilized and stained with rhodamine-labelled phalloidin for actin filaments according to a commercial protocol (Molecular probe). Cells were observed under a fluorescence microscopy, equipped with a high content analysis (HCA) and high content screening (HCS), by an IN Cell Analyzer 2000 (GE Healthcare; excitation at 495 nm, and emission at 520 nm).

### Endothelial cell migration assays

Endothelial cell migration was assayed with scrape assays. Briefly, ERα or ROCK-II siRNA of endothelial cells were transferred in 12 well plates for 48 h and synchronized by replacing the medium with human endothelial serum-free medium (ATCC), devoid of growth factors for 12 h. The HUVEC were then scraped away horizontally in each well using a P100 pipette tip. Three randomly-selected views along the scraped line were photographed on each well using phase-contrast microscopy. The media was immediately replaced with fresh media in the presence or absence of formononetin. Following 16 h of incubation, a second set of images was photographed. To determine whether HUVEC had migrated, the images were analyzed using the Metamorph Imaging Series software package. The mean length of the scraped area in each condition was measured and deducted from the value obtained prior to treatment. The change in length of the experimental condition was compared with that of the control (0.1% DMSO). A decrease in the mean length of the scraped area indicated that HUVEC had migrated.

### Cell real-time migration assay

Real-time monitoring of endothelial cell migration was performed using the xCELLigence system with the CIM-Plate 16 (Roche). The upper chamber was coated with 20 μg/ml Fibronectin (BD Biosciences) and seeded with ERα or ROCK-II siRNA-transfected at 50,000 HUVECs. When cells migrated through the membrane into the bottom chamber in response to attractants, they contacted and adhered to the electronic sensors, resulting in increased impedance. The cell-index values reflecting impedance changes were automatically and continuously recorded every 15 min.

### Immunoblotting

HUVECs were treated with 50 μM formononetin for different time durations (between 5 and 60 min) during the time course study. Medium with 0.1% DMSO served as a vehicle control. For inhibition assays, HUVECs were pretreated with 10 μM ICI182, 780 for 60 min prior to the addition of 50 μM formononetin. Cells were then washed with PBS and lysed for 20 min on ice with lysis buffer (0.5 M NaCl, 50 mM Tris, 1 mM EDTA, 0.05% SDS, 0.5% Triton X- 100, 1 mM PMSF, pH 7.4). Cell lysates were centrifuged at 12,000 g for 20 min at 4 °C. Protein concentrations in the supernatants were measured using the bicinchoninic acid assay (Pierce, Rockford, IL). Supernatants were electrophoresed on 12% SDS-PAGE, and transferred to polyvinylidene diuoride (PVDF) membranes, which were subsequently blocked with 5% non-fat milk. Antibodies against the following proteins were used: MMP2/9, P-MYPT1 853/MYPT1, P-cofilin/cofilin, P-MLC2/MLC2, ROCK-II, ERα and beta-actin. After the secondary antibodies were incubated for 1 h, proteins were detected using an enhanced ECL system (GE Healthcare, Little Chalfont, Buckinghamshire, UK). Semi-quantifications were performed according to densitometric analysis, in conjunction with the Quantity One software package.

### Coimmunoprecipitation Assays

HUVEC were harvested in 100 mM Tris-HCl (pH 6.8), 4% sodium dodecyl sulfate (SDS), 20% glycerol, 1 mM Na3VO4, 1 mM NaF, and 1 mM phenylmethylsulfonyl fluoride (PMSF). Equal amounts of cell lysates were incubated with 1 μg precipitating Ab, overnight at 4 °C under gentle agitation. 25 μl of a 1:1 ChIP-Grade Protein G Agarose Bead (Cat. no. 9007 s, Cell Signaling Technology) were added, and the samples were rolled at 4 °C for 3 h. The samples were then pelleted, washed, and resuspended in 25 μl 2 × Laemmli buffer for immunoblotting.

### Statistical analysis

Each experiment was performed independently at least 3 times. Means ± *SD* were compared using the Student’s *t*-test according to the following statistical criteria: *p* < 0.05 = significant; *p* < 0.01 = highly significant; *p* < 0.001 = extremely significant. All relevant data are presented as means ± *SD*.

## Additional Information

**How to cite this article**: Li, S. *et al*. Formononetin promotes angiogenesis through the estrogen receptor alpha-enhanced ROCK pathway. *Sci. Rep*. **5**, 16815; doi: 10.1038/srep16815 (2015).

## Supplementary Material

Supplementary Information
