# Supplementary material for: Formononetin promotes angiogenesis through the estrogen receptor alpha-enhanced ROCK pathway
Source: Sci Rep. 2015 Nov 16;5:16815. doi: 10.1038/srep16815 (PMC4645220; doi:10.1038/srep16815)
Supplement: Supplementary Information [file srep16815-s1.doc]

**Formononetin promotes angiogenesis through the estrogen receptor alpha-enhanced ROCK pathway**

Shang Li ^1^, Yuanye Dang ^1^, Xuelin Zhou^2^, Bin Huang ^1^, Xiaohui Huang ^1^, Zherui Zhang ^1^, Yiu Wa Kwan ^2^, Shun Wan Chan ^3^, George Pak Heng Leung ^4^, Simon Ming Yuen Lee ^1, *^, Pui Man Hoi ^1, *^

^1^ State Key Laboratory of Quality Research in Chinese Medicine and Institute of Chinese Medical Sciences, University of Macau, Macao, China

^2^ School of Biomedical Sciences, Faculty of Medicine, The Chinese University of Hong Kong, Shatin, N.T., Hong Kong, China

^3^ State Key Laboratory of Chinese Medicine and Molecular Pharmacology, Department of Applied Biology and Chemical Technology, The Hong Kong Polytechnic University, Hong Kong, China

^4^ Pharmacology and Pharmacy, Faculty of Medicine, The University of Hong Kong, Hong Kong, China

*Corresponding authors at: State Key Laboratory of Quality Research in Chinese Medicine and Institute of Chinese Medical Sciences, University of Macau. Av. da Universidade, N22 Building. Taipa, Macau. Fax: +853 88221358. E-mail addresses: simonlee@umac.mo, maghoi@umac.mo.

**Supplementary Table and Figures**


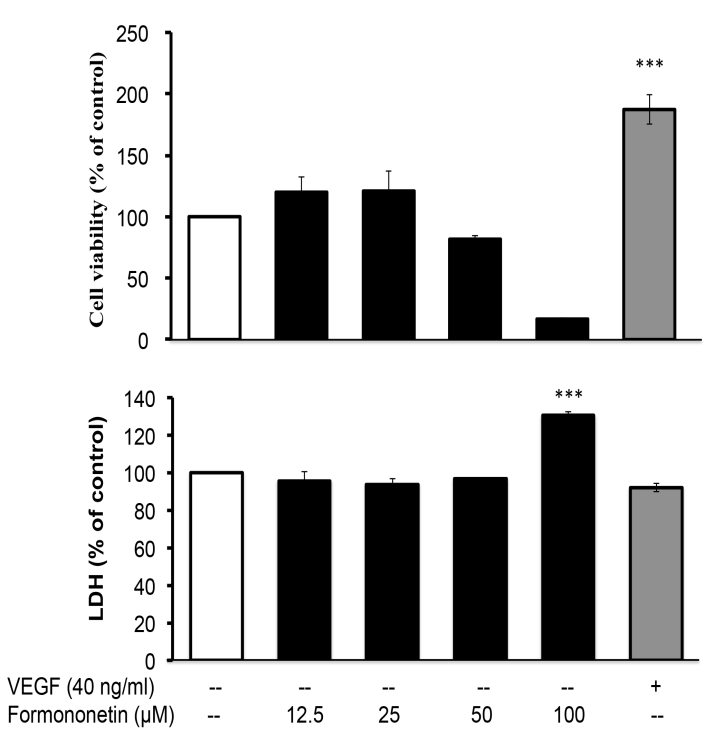


**SFigure 1. Effects of formononetin on cell viability and cytotoxicity of HUVEC were detected with XTT and LDH assay**.

Starved HUVECs were treated with formononetin for 24 (LDH) or 48 (XTT) hours. Among them, treatment of 0.5% FBS or 20ng/ml VEGF were served as vehicle control and positive control, respectfully. Results are expressed as percentage of controls (mean ± *SD*; *n* > 3), ****p <0.001 vs.* control.


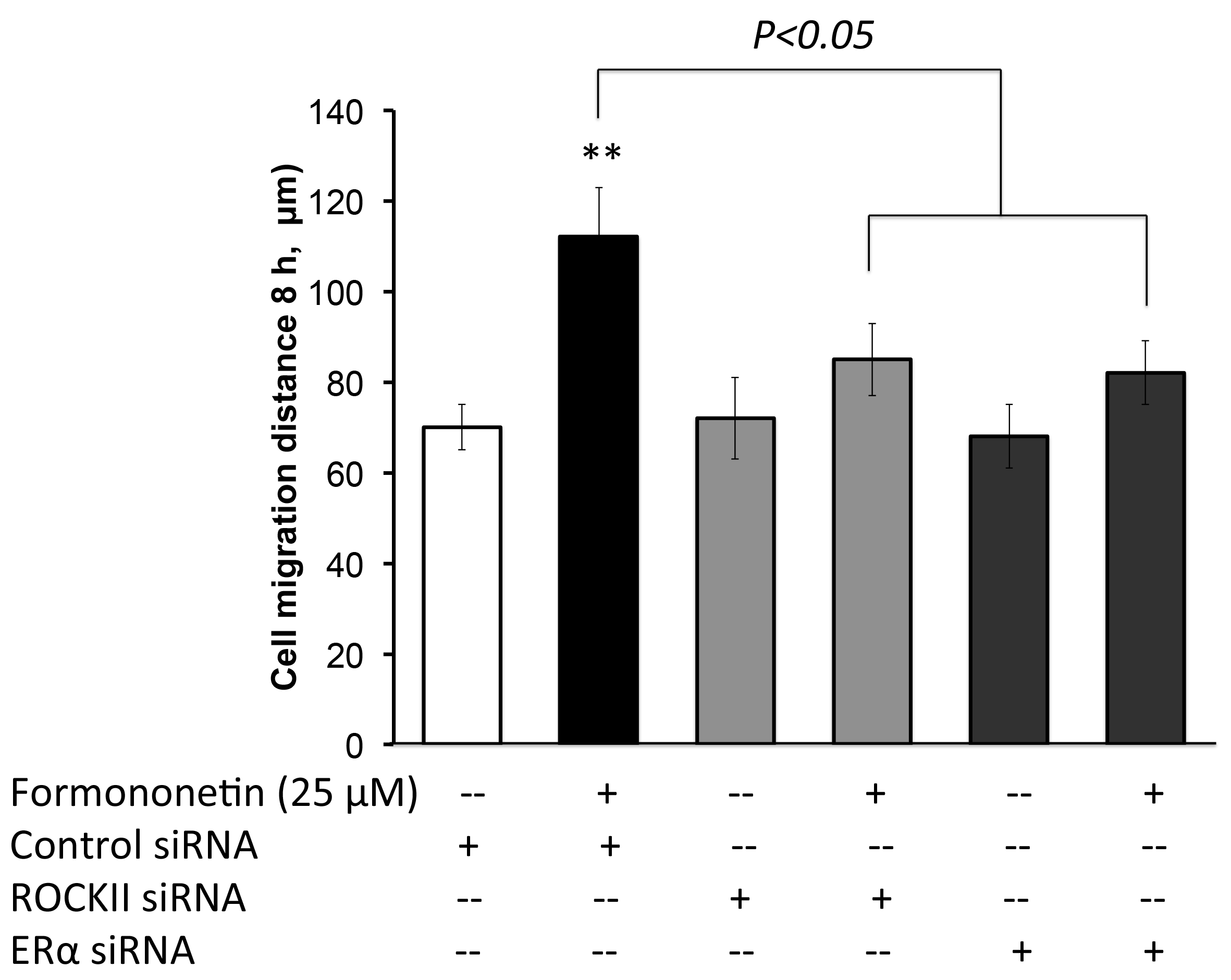


**SFigure 2. ERα or ROCK-II blockade inhibited formononetin-induced HUVECs cell migration**.

Wound healing assay. Transfected HUVEC cells (control siRNA, ROCK-II siRNA, ERα siRNA) were grown to 80-90% confluence in a 24-well plate, and incubated in medium containing 1% FBS overnight. HUVEC monolayers were scratched and cultured in medium containing 2% FBS, to facilitate cell migration. Cells were treated with 2% FBS medium or 25 µM formononetin for 8 h. Cell migration was recorded by phase contrast microscopy following wound scratch. The extent of cell migration into the wound scratch was quantified as the wound healing distance, and compared to that of control cells at 8 h. **p < 0.05*, ***p < 0.01* *vs*. control group. Values represented the means ± *SD* of three independent measurements along the wound scratch.

A) VEGFR2


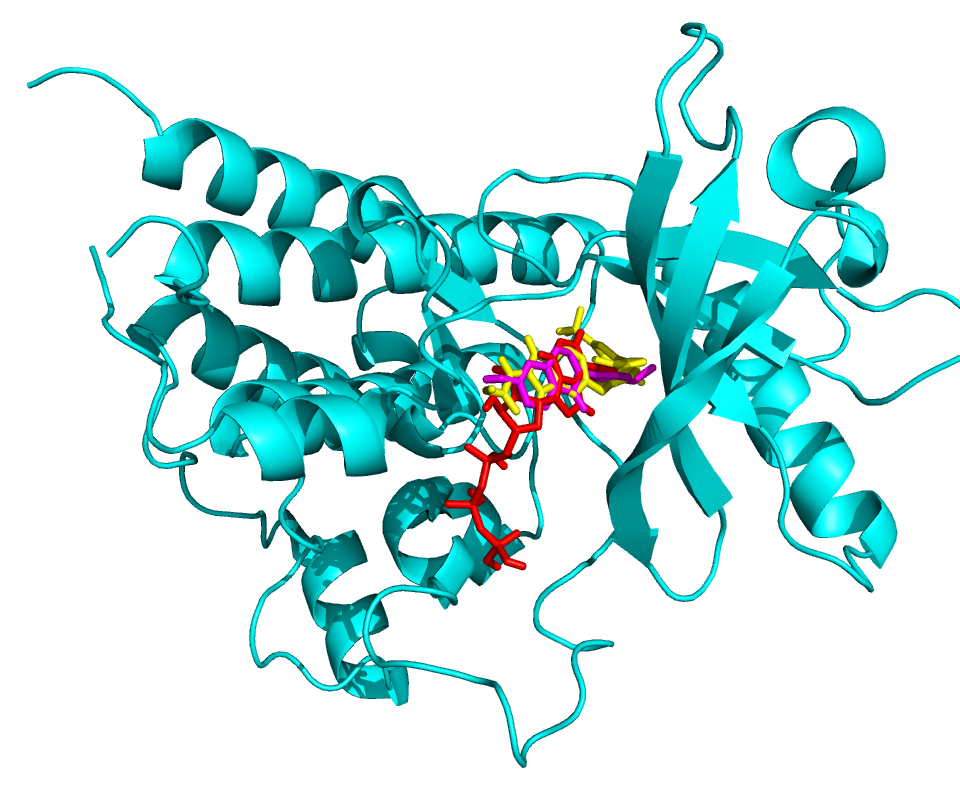


B) TGF-beta receptor 1


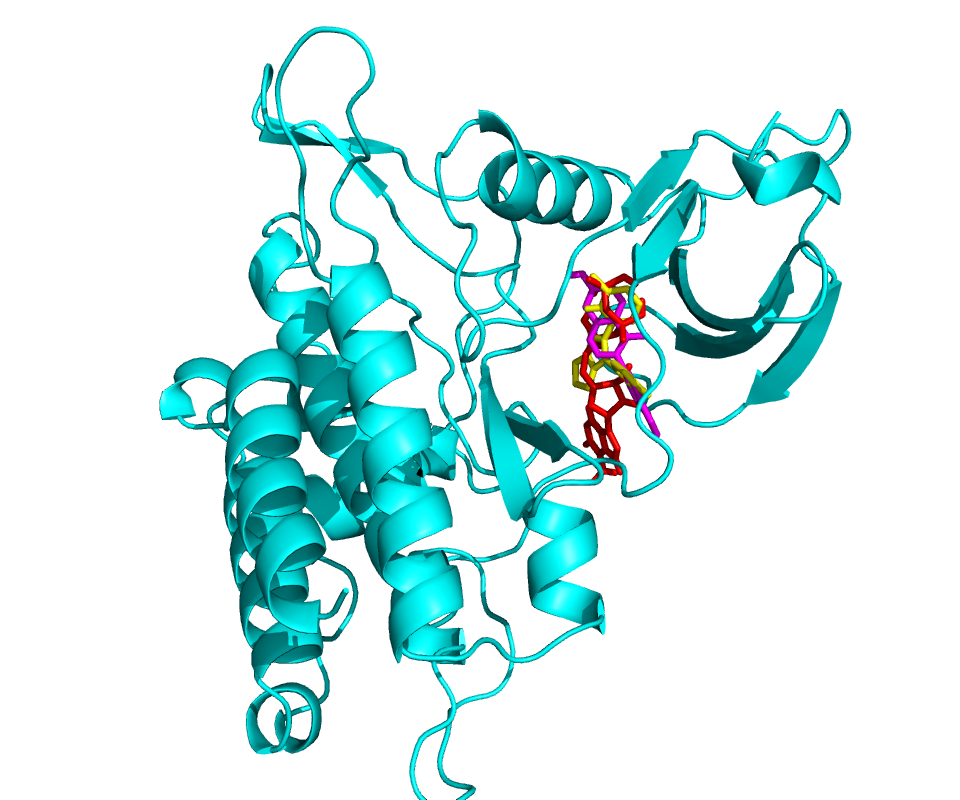


C) RhoA


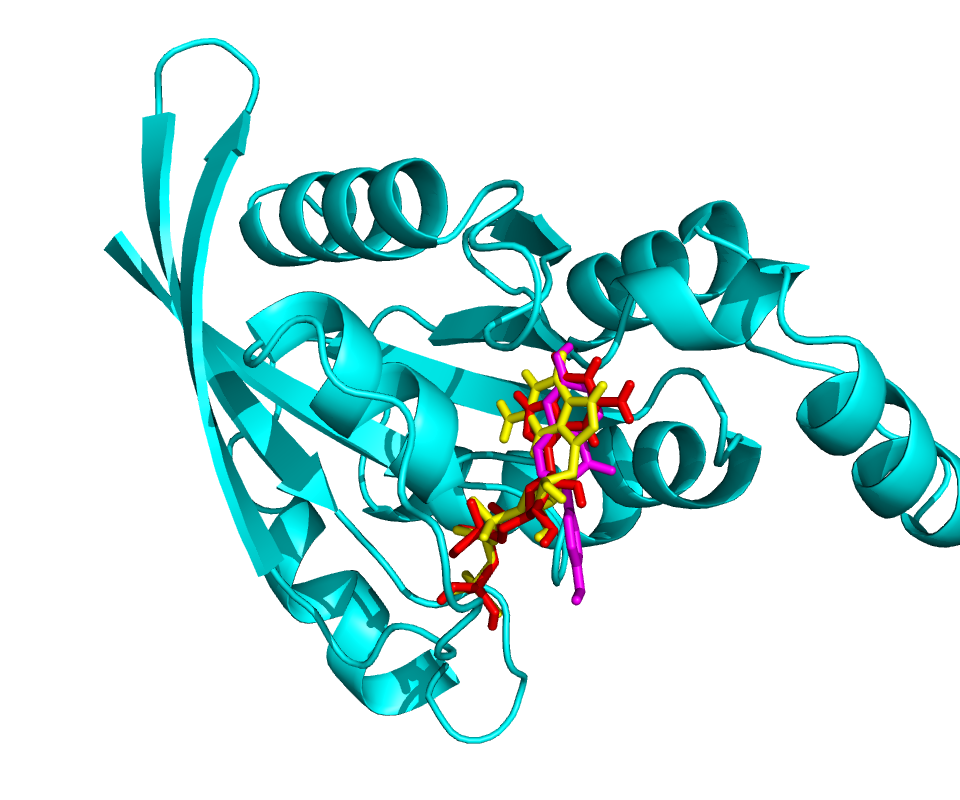


**SFigure 3.** Binding conformation of formononetin and ATP or GTP to the ATP/GTP binding site of human VEGF receptor 2 (A; PDB ID 2OH4), TGF-beta receptor 1 (B; PDB ID 1RW8) and RhoA (C; PDB ID 1A2B). Positive control: yellow; ATP/GTP: red; Formononetin: meganta

| Receptor/kinase | PDB ID | Ligand | Binding free energy |
| --- | --- | --- | --- |
| VEGFR2 | 2OH4 | ATP | -8.4 |
|  |  | Formononetin | -9.2 |
|  |  | GIG^*^ | -13 |
| TGF-beta receptor 1 | 1RW8 | ATP | -8.7 |
|  |  | Formononetin | -8.8 |
|  |  | 580^*^ | -10.8 |
| RhoA | 1A2B | GTP | -10.3 |
|  |  | Formononetin | -7.1 |
|  |  | GSP^*^ | -10.3 |

*: positive control in each receptor/kinase.

**STable 1.** Logarithm of binding free energies (kcal/mol) of formononetin to the ATP binding sits of human VEGF receptor 2 (PDB ID 2OH4), TGF-beta receptor 1 (PDB ID 1RW8) and RhoA (PDB ID 1A2B). GIG: Methyl (5-{4-[({[2-fluoro-5-(trifluoromethyl)phenyl]amino}carbonyl)amino] phenoxy}-1H-benzimidazol-2-yl)carbamate;

580: 3-(4-fluorophenyl)-2-(6-methylpyridin-2-yl)-5,6-dihydro-4h-pyrrolo[1,2-b] pyrazole

GSP: 5'-Guanosine-diphosphate-monothiophosphate

**Supplementary Results**

As shown in Supplemental Figure 3, formononetin and ATP/GTP can bind to the ATP/GTP binding site of these three proteins: VEGFR2 (A), TGF-beta receptor 1 (B), or RhoA (C). According to their binding free energies as shown in Supplemental Table 1, formononetin can very weakly bind to the ATP binding sites of VEGFR2 and TGF-beta receptor 1 when compared with the binding affinity of ATP. For RhoA, formononetin failed to compete with GTP to bind to the GTP binding site of RhoA. Meanwhile, the positive controls strongly bound to the ATP/GTP binding sites of these receptors. Through the docking analysis, formononetin strongly bound to LBD of ERalpha, weakly bound the kinase activation site VEGFR2 and TGF-beta receptor 1, but did not bind to RhoA. These results indicated that formononetin induced the activation of ROCK signaling pathway via the interaction with ERα.

**Supplementary Methods**

**Molecular docking study**

Molecular docking of formonoetin to receptor(s)/kinase(s) was performed using the three dimensional (3-D) crystal structures of human VEGF receptor 2 (PDB ID 2OH4), TGF-beta receptor 1 (PDB ID 1RW8) and RhoA (PDB ID 1A2B) which were obtained from the Protein Data Bank. The software AutoDock Vina v.1.0.2 was used for docking. Briefly, the docking parameters for AutoDock Vina were kept as their default values. The grid box was 20 Å × 20 Å × 20 Å, encompassing the ATP binding sites of the kinase domain. The binding modes were clustered through the root-mean square deviation (RMSD) among the Cartesian coordinates of the ligand atoms. The docking results were ranked by the binding free energy, and the binding modes with lowest binding free energy were chosen as the optimum docking conformation. The binding conformations were simulated by PyMOL Molecular Graphics System Version 1.3 (Schrödinger, OR, USA).

**Supplementary References**

1. Hasegawa M, Nishigaki N, Washio Y, Kano K, Harris PA, Sato H, et al. Discovery of novel Benzimidazoles as potent inhibitors of TIE-2 and VEGFR-2 tyrosine kinase receptors. J Med Chem. 2007 Sep 6; 50(18):4453-70.

2. Sawyer JS, Beight DW, Britt KS, Anderson BD, Campbell RM, Goodson T, et al. Synthesis and activity of new aryl- and heteroaryl-substituted 5,6-dihydro-4H-pyrrolo[1,2-b]pyrazole inhibitors of the transforming growth factor-beta type I receptor kinase domain. Bioorg Med Chem Lett. 2004 Jul 5; 14(13):3581-4.

3. Ihara K, Muraguchi S, Kato M, Shimizu T, Shirakawa M, Kuroda S, et al. Crystal structure of human RhoA in a dominantly active form complexed with a GTP analogue. J Biol Chem. 1998 Apr 17; 273(16):9656-66.

4. Seeliger D, de Groot BL. Ligand docking and binding site analysis with PyMOL and Autodock/Vina. J Comput Aided Mol Des. 2010 May; 24(5):417-22.
